# A Short Message Service Intervention for Improving Infant Feeding Practices in Shanghai, China: Planning, Implementation, and Process Evaluation

**DOI:** 10.2196/11039

**Published:** 2018-10-29

**Authors:** Hong Jiang, Mu Li, Li Ming Wen, Louise A Baur, Gengsheng He, Xiaoying Ma, Xu Qian

**Affiliations:** 1 Global Health Institute School of Public Health Fudan University Shanghai China; 2 Key Laboratory of Public Health Safety, Ministry of Education Fudan University Shanghai China; 3 School of Public Health University of Sydney Sydney Australia; 4 China Studies Centre University of Sydney Sydney Australia; 5 Health Promotion Unit Sydney Local Health District Sydney Australia; 6 Discipline of Child & Adolescent Health University of Sydney Sydney Australia

**Keywords:** mHealth, short message service, breastfeeding, infant feeding practices, health services, planning and development, implementation, process evaluation

## Abstract

**Background:**

Although mobile health (mHealth) has been widely applied in health care services, few studies have reported the detailed process of the development and implementation of text messaging (short message service, SMS) interventions.

**Objective:**

Our study aims to demonstrate the process and lessons learned from a community-based text messaging (SMS) intervention for improving infant feeding in Shanghai, China.

**Methods:**

The intervention included planning and development, implementation, and process evaluation. A 3-phase process was adopted during planning and development: (1) a formative study with expectant and new mothers to explore the barriers of appropriate infant feeding practices; (2) a baseline questionnaire survey to understand potential intervention approaches; and (3) development of the text message bank. The text messaging intervention was delivered via a computer-based platform. A message bank was established before the start of the intervention containing information on the benefits of breastfeeding, preparing for breastfeeding, early initiation of breastfeeding, timely introduction of complementary foods, and establishing appropriate feeding practices, etc. An expert advisory committee oversaw the content and quality of the message bank. Process evaluation was conducted through field records and qualitative interviews with participating mothers.

**Results:**

We found that the text messaging intervention was feasible and well received by mothers because of its easy and flexible access. The weekly based message frequency was thought to be appropriate, and the contents were anticipatory and trustworthy. Some mothers had high expectations for timely response to inquiries. Occasionally, the text messages were not delivered due to unstable telecommunication transmission. Mothers suggested that the messages could be more personalized.

**Conclusions:**

This study demonstrates the feasibility and value of text messaging intervention in filling gaps in delivering health care services and promoting healthy infant feeding practices in settings where personal contact is limited.

## Introduction

The Life Course Health Development Model suggests that health interventions should focus on early childhood to build a solid basis for long-term health [1]. Infant feeding practices may have long-term effects by influencing eating behaviors and obesity risk in later life [2-6].

Inappropriate infant feeding knowledge and practices have been widespread among new parents or caregivers. Examples include the perception that the nutritional value of infant formula is higher than breastmilk, too long or too short feeding intervals, providing few varieties of fruit, no chance for the baby to learn eating, and using food as a reward [7-10]. Parents are the “gatekeepers” of children’s eating environments. Their feeding practices strongly influence children’s eating patterns, which lay the foundation of future eating habits and children’s weight [11,12]. Research has shown that rapid early weight gain before 2 years of age is associated with the increased risk of later overweight or obesity, and infant feeding practices have a high potential for long-term health effects [13-15]. Undoubtedly, first-time mothers are in greater need of guidance and support on appropriate infant feeding practices.

Mobile phone ownership and subscription have been rising sharply in the past decade, including in developing countries such as India and China. Globally, there are approximately 7.6 billion mobile phone subscriptions, growing 4% year-on-year [16]. The application of mobile phones in delivering health care, known as mobile health (mHealth), has attracted much attention [17,18]. Short message service (SMS) text messaging, as an example of mHealth, represents an innovative channel for information to be delivered cheaply to people wherever they are located and whenever available [19]. In recent years, the use of SMS text messaging for health service delivery and public health interventions has also been on the rise [20-22]. Despite the rapid growth and application of mHealth, including SMS text messaging, in public health, to date, few studies have detailed the experience and lessons learned from planning, development, and implementation of SMS text messaging interventions. The aim of this paper is to present the planning and development, implementation, and process evaluation of a community-based SMS text messaging intervention targeted at improved infant feeding practices in Shanghai, China.

## Methods

### Short Message Service Text Messaging Intervention Design

A 3-year community-based SMS text messaging intervention research was developed and implemented in Shanghai to explore an innovative approach to support new mothers for establishing appropriate infant feeding practices in an effort to prevent childhood obesity. The study is a quasi-experimental design. We purposively selected 4 community health centers (CHCs) from 2 districts of Shanghai. In each district, 2 CHCs with a similar population size were selected; one CHC was randomly assigned as the intervention and the other as the control site. Therefore, 2 CHCs were assigned as intervention group and 2 as control group. During the first antenatal visit to the CHCs in the first trimester, pregnant women who gave consent were recruited for the study. Before the third trimester, the pregnant women recruited for the study were contacted to confirm their eligibility and willingness to participate in the study. Participants in the intervention group would then receive weekly SMS text messages on breastfeeding and infant feeding from the third trimester to 12 months postpartum in addition to the usual health care they received from CHCs. Participating mothers in the control group received the usual health care services during late pregnancy and postpartum, as did the intervention group. Infants of mothers in both groups had the same routine physical checkups at the CHCs in the first year. The median duration of exclusive breastfeeding (EBF) and the prevalence of EBF at 6 months were the main outcome measures and were compared between the two groups [23]. Other outcomes included any breastfeeding rate at 12 months and some infant feeding practices (eg, food for reward, etc). In this paper, we report the process of the planning and development, implementation, and process evaluation of the intervention using documentation such as researchers’ diaries, field records, project meeting minutes, and qualitative interviews with participants. The study was conducted between September 2010 and December 2013.

### Intervention Planning and Development

The development of this SMS text messaging intervention was informed by the Healthy Beginnings Trial, which was a staged, home-based, early obesity intervention in the first 2 years of life [24]. However, the home visiting approach is a very costly approach that may not be feasible in the Chinese context. With emerging evidence regarding the use of SMS text messaging in health promotion interventions, we considered whether SMS text messaging could act as an alternative means to deliver staged early interventions to mothers of young children, at a lower cost and with greater population reach.

In planning and developing the SMS text messaging intervention, we adopted a 3-phase process, including: (1) qualitative interviews with pregnant women and new mothers to explore barriers in appropriate infant feeding practices; (2) a baseline questionnaire survey to understand the preferred intervention approaches among pregnant women and new mothers; and (3) the development of an SMS text message bank.

#### Phase 1: Qualitative Interviews to Explore the Barriers

The initial qualitative study aimed to: (1) enquire about any existing infant feeding-related interventions the women have received from the health system during perinatal care; (2) explore barriers to EBF and the reasons preventing women from breastfeeding for the recommended duration and to elicit any perceived difficulties in introducing complementary foods to infants; (3) identify culturally appropriate ways to access and interact with pregnant women and new mothers; and (4) explore the preferred frequency and time for receiving messages.

We conducted in-depth interviews with 24 new mothers who had infants younger than 12 months of age and conducted two focus groups, each with 7 pregnant women in the late third trimester. Methods of data collection and analysis have been published elsewhere [25]. We found that: (1) there was limited communication between health professionals and either pregnant women or new mothers about breastfeeding; (2) there was inadequate support from health professionals for dealing with breastfeeding difficulties and problems of infant feeding; (3) the participants thought that the mobile phone could potentially be an effective channel of communication between themselves and health professionals; and (4) the preferred frequency and time of receiving messages were on a weekly basis and during daytime (from 9:00 am to 6:00 pm) [26].

#### Phase 2: Baseline Questionnaire Survey

A baseline survey was carried out in the 4 communities selected for the intervention study. A total of 653 pregnant women who consented to join in the SMS text messaging intervention were invited to participate in the baseline survey during their first visit to the prenatal care service in the CHCs before 22 weeks’ gestation. The survey results showed that (1) awareness of World Health Organization (WHO) breastfeeding guidelines among these women was low; (2) intention to breastfeed exclusively during the first 6 months among these women was also low; and (3) nearly all women owned mobile phones, and around 94.6%(618/653) of women continued to use mobile phones during their pregnancy [25,26].

#### Phase 3: Development of a Message Bank

The above results were used to inform the development of an SMS text message bank and intervention model. A message bank was established before the start of the intervention and evolved during the project. The information source included the WHO Breastfeeding Guidelines [27], the Chinese Residents Nutrition Guidelines [28], and recommendations from the infant feeding literature, through consultations with pediatricians and community child health workers as well as findings from the formative qualitative study and baseline survey. The key information on infant feeding was edited to be suitable for delivery via an SMS text message. The guiding principles for designing messages included consideration of key knowledge of infant feeding, use of an evidence-based approach, use of simple language, avoidance of technical terminology, and a supportive and friendly tone. Each message usually covered one issue or topic and was between 180 to 210 characters, allowing it to be shown on one mobile phone screen as a complete message.

The research team, including researchers in maternal and child health areas from both Fudan University, China, and the University of Sydney, Australia, designed the message bank. An expert advisory committee was established to provide guidance and review the contents of breastfeeding and infant feeding messages for children aged 0-12 months of age. The advisory committee included pediatricians and child health care professionals from municipal, district, and community levels and academic researchers of maternal and child health care in China.

### The Implementation of the Short Message Service Intervention

The intervention was implemented in 4 purposively selected CHCs from 2 administrative districts of Shanghai [23]. Of these, 2 CHCs were assigned as the intervention group (n=281), and the other 2 were the control group (n=301). All participants were first-time mothers. The intervention group received a weekly message relating to appropriate infant feeding practices via their mobile phone as well as routine perinatal and child health care. Participating mothers in the control group only received the routine perinatal and child health care services.

#### Platform for Short Message Service Text Messaging Delivery

**Figure 1:**
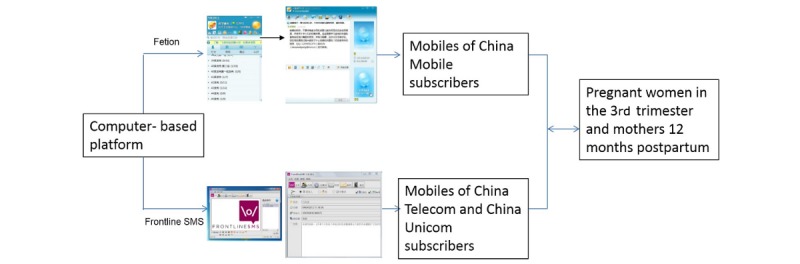
The short message service (SMS) text messaging operation platform.

Messages were sent from 2 free computer-based platforms: (1) “Fetion” for sending messages to China Mobile subscribers, that is, >90% of the study participants, with no cost for sending or receiving messages and (2) “Frontline SMS” for sending messages to subscribers of China Telecom and China Unicom, which cost 0.1 renminbi per message (≈US $0.017 ), but there is no cost for receiving messages (Figure 1). The research team at Fudan University was responsible for sending messages to participants through the platforms.

**Examples of short message service (SMS) text messages for infant feeding (translated from Chinese to English).**

**Late pregnancy (1st message in the 3rd trimester)**

- To all mothers:

*Dear Mom, congratulations! You are now entering the third trimester. In 2-3 months, your baby will be born. Have you started thinking about the way you will give birth and feed your new baby? From now on, we will help you with these issues. World Health Organization recommends that exclusively breastfeeding baby in the first 6 months can help achieve optimal growth, development, and health. From next week, we will send information to you through SMS text message. You are welcome to contact us by sending us SMS text messages or call us directly!*

**The 4th week after birth**

- To mothers practicing exclusive breastfeeding:

*Dear Mother, your baby is going to enter the 2nd month. In this month, your baby will be able to raise his or her head. Some mothers would ask whether they should add formula to baby’s milk to meet the baby’s growth needs. Your breast milk can meet all nutritional needs of your baby before 6 months. We strongly suggest you to exclusively breastfeed in the first 6 months. This means breastfeeding without adding any other food or drink, including water, since water is the main component of breast milk.*

- To mothers practicing mixed breastfeeding or formula feeding:

*Dear Mother, for whatever reasons you cannot exclusively breastfeed your babies, it is recommended that you feed your baby with infant formula for appropriate age. Please follow the instructions for the appropriate quantity of water. Baby could have diarrhea if the formula is too concentrated. If the formula is too thin, it can cause malnutrition. Please apply hygienic practice when handling formula. Leftover milk should be disposed.*

#### Content Delivered

The SMS text messaging intervention was delivered from the beginning of the third trimester (28 weeks gestation) to 12 months postpartum (ie, 66 weeks in total). Before the intervention commenced, mothers in the intervention group were sent a greeting message, alerting them to the beginning of the intervention with a brief introduction of the project. This was followed by the first weekly intervention message.

The SMS text messages were tailored initially for mothers in the third trimester of pregnancy and then for the different stages of child development in the first 12 months. For example, during the third trimester, key messages were focused on the benefits of breastfeeding for both babies and mothers and on advising mothers to be mentally and physically prepared for breastfeeding. Within the first week of giving birth, the emphasis was on helping mothers with different delivery modes to initiate breastfeeding. There were 3 sets of messages according to mothers’ breastfeeding and work status after childbirth [23]. The corresponding messages were sent once the infant feeding practices changed. SMS text messages were also used for collecting information on infant feeding status from mothers to trigger the delivery of appropriate messages. From the 35th gestational week, in addition to weekly messages about specific content, separate messages were also sent to mothers inquiring whether they had given birth, their breastfeeding status, whether they had returned to work, or the timing of introduction of solids to determine the relevant types and content of messages to be sent. For any other conditions, for example, if the infant became sick, mothers were encouraged to contact the research team through SMS text messages and to seek medical attention. Routine health check-up records in CHCs were also reviewed to obtain or confirm information on the change in infant feeding practices.

Since most Chinese women return to work around 4 months after childbirth, from the 3rd month, specific messages were sent to the mothers who would go back to work, encouraging them to continue EBF. At the 4th and 5th month postpartum, mothers were advised how to introduce complementary foods from the 6th month and how to establish appropriate feeding practices. Tactics and strategies on how to deal with problems during infant feeding in the next several months were also suggested (examples are shown in Textbox 1).

## Results

In this paper, we report the results of the intervention process evaluation. The effect of the intervention on breastfeeding practices has been previously reported [23]. Briefly, compared with the control group, the intervention group had a significantly longer median duration of EBF at 6 months (11.41 weeks, 95% CI 10.25-12.57 vs 8.87 weeks, 95% CI 7.84-9.89). The intervention resulted in a significantly higher rate of EBF at 6 months (adjusted odds ratio=2.67, 95% CI 1.45-4.91) [23].

The process evaluation was conducted across the entire duration of the intervention and included: (1) records of questions received from mothers via SMS text messages, records of mothers’ reports on changes in feeding practices, and records of the number of messages sent; (2) qualitative interviews with mothers in the midterm of the intervention; and (3) qualitative interviews with mothers at the end of the intervention.

The rate of feedback from mothers on the status of infant feeding through messages was 73.0% (205/281) and 60.4% (160/265) at the end of the 1st and 4th postnatal months, respectively. By the end of the intervention, a total of 19,108 messages had been sent, an average of 68 messages to each participant.

While we delivered weekly SMS text messages to mothers, they were encouraged to ask questions and communicate with the research team about problems and concerns encountered during infant feeding. If the research team received inquiries about breastfeeding or complementary feeding, we would either respond directly or consult members of the expert committee.

If inquiries were about a medical condition, we would suggest mothers seek medical attention. By the end of the intervention, 43.4% (122/281) of mothers in the intervention group made inquiries through SMS text messages. A total of 601 messages were received from the participants. These inquiries covered three main areas:

1. Breastfeeding (eg, insufficient milk supply, reflux, baby falling asleep while breastfeeding, breastfeeding while the mother had an upper respiratory tract infection, dietary restrictions, approaches to expressing milk, dealing with mastitis)
2. Introduction of complementary food (eg, when and how much of the calcium and vitamin A supplement to give)
3. Other issues (eg, neonatal jaundice, eczema, diarrhea, upper respiratory tract infections, sucking hand, etc)

A midterm evaluation was conducted through in-depth interviews with mothers 6-8 months after the start of the intervention to obtain feedback on whether the intervention was implemented as designed and whether the intervention frequency and content were appropriate. A total of 22 mothers with various infant feeding practices (EBF, mixed breastfeeding, infant formula feeding) were purposively selected by the CHCs. Mothers were asked about their overall reflection on the intervention and were prompted for issues emerging from the intervention process.

Table 1 shows the themes identified from the interviews. There was positive feedback from mothers on the intervention frequency, content, feedback to questions, and the suitability of the language level of SMS text messaging intervention. Concerns that emerged from the interviews included occasional failure of receiving messages in one residential area due to an unstable telecommunication signal and unmet expectations in the time to respond to questions. In addressing these issues, a follow-up message was sent to all mothers in that area, asking whether they had received a message in the past 7 days. In addition, mothers were encouraged to inform the research staff if they did not receive the scheduled message. The research team also developed a protocol to ensure that all inquiries should be replied to within the same working day. If the inquiries were made at night or weekends, they would be replied to on the following morning or the following Monday morning.

At the completion of the intervention, in-depth interviews were carried out with 15 mothers in the intervention group to elicit perspectives of service users on the strengths and limitations of the SMS text messaging intervention. Interviewees were purposely selected based on their different types of infant feeding practices and mothers’ work status (returned to work or still staying at home). The perceived strengths of the SMS text messaging intervention included convenient and information can be accessed repeatedly, information could be shared with friends and family members, timely support and anticipatory guiding, and trust to the information as it came from a trustworthy source. The main perceived weakness of the SMS text messaging in this study included the limited information load because of the word limit in a message and lack of personalized contents (Table 1).

**Table 1:** Themes and selected quotes from the process evaluation.

Themes			Selected quotes
**Midterm evaluation**			
	**Positive reflections on short message service (SMS) text messaging intervention**		
		Timely delivery in most intervention areas	*[I remember receiving messages] once a week…sometimes two to three messages in the week [including messages inquiring BF^a^ status from research group].* [EBF^b^, 4 months postpartum]
		Acceptable and appropriate intervention frequency and intensity	*I feel the weekly message is good. I feel this frequency can cover all information of my needs during the week.* [EBF, 5 months postpartum]
		Anticipatory and appropriate SMS text messaging contents	*The messages were sent timely, [the contents are] always a bit earlier than what will happen.* [EBF, 4 months postpartum] *I learnt not to feed sugar water to baby in one message. My parents used to feed my baby sugar water. But since I received the message, we do not give it anymore.* [Mixed infant feeding, less than 1 month postpartum]
		Replying to questions being helpful in dealing with problems of infant feeding	*I made an inquiry of what to do with inadequate breast milk after returning to work through the message. I felt the response was very helpful.* [EBF, 5 months postpartum]
	**Problems emerging at midterm evaluation**		
		The occasional failure of message sending in one neighborhood due to unstable mobile signal in the area	*It seems every a couple of weeks [for receiving messages]…the telecom signal is not very good in my house area.* [Mixed infant feeding, 4 months postpartum]
		High expectation on timely response to inquiries	*Sometimes I got responses very quickly, but I received the message on the following day if I sent the question at night.* [Having stopped breastfeeding, 4 months postpartum]
**Final qualitative evaluation**			
	**Strength of SMS text messaging**		
		Convenient to save, read, repeatedly review, and share with others	*I saved all messages. And usually I shared them with my mother-in-law…Sometime she does not agree with me on some infant feeding practice, so I show the message to her. For example, she wanted to feed egg to the baby at 4 months, I declined, but she continued to try and said she already fed my husband when he was 4 months old. But after I showed the message to her, she then stopped.* [BF to 10 months, returned to work at 4 months]
		Timely support and anticipatory guiding	*To me, messages allow me continuously learn about [infant feeding]. The contents [of messages] match the age of my baby, and they can be applied to the practice soon.* [EBF to 6 months, BF to 9 months, return to work 14 months postpartum]
		Easy to build the trust	*I feel the messages like a friend of mine. I do not go to the internet to search for specific information now. I am accustomed to receiving the weekly message and feel this is a very natural thing.* [EBF to 6 months, returned to work at 4.5 months]
	**Weakness of SMS text messaging**		
		Limited information due to the word limit of messages	*I feel messages could be a reference for me, but the content is not long enough.* [Infant formula feeding, returned to work at 6.5 months]
		Messages could have been more “personalized”	*I feel the messages were similar to those on the internet and books. I would prefer information that could not be found in the internet, particularly on how to let my baby eat more.* [BF to 9 months, returned to work at 4 months]

^a^BF: breastfeeding.

^b^EBF: exclusive breastfeeding.

## Discussion

In this paper, we have documented the planning and development, implementation, and process evaluation of an SMS text messaging intervention aimed at improving infant feeding practices. Given the limited literature on the detailed process of mHealth interventions, this paper contributes to informing and improving the development and implementation of future mHealth interventions and infant feeding research.

The strengths of the study are 2-fold. First, the study took a systematic approach based on the health promotion management framework [29], including planning and development, implementation, and process evaluation. Second, the development of the intervention was evidence based. The overall intervention strategy was to establish a supportive environment for mothers, which was informed by the needs assessment from the qualitative interviews during intervention planning and development. For example, we adopted mothers’ preferences of receiving a weekly push message rather than a higher frequency. This was also indicated by two previous mHealth studies that showed behavior changes were significantly better among patients who received a weekly supporting message when compared to those with a daily reminding message [30-32].

The implementation of the SMS text messaging intervention used a computer-based device that was easy to operate, even in resource-limited settings. The process evaluation revealed some positive qualities of the study. Mothers viewed the SMS text messaging intervention favorably as they enjoyed the easy and flexible access in terms of cost and time as well as the ability to view SMS text messages repeatedly and share with others. In addition, mothers felt confident about applying practices promoted in messages from trustworthy sources, for example, health care service providers or public health researchers. This indicates that information from trusted sources is important for the SMS text messaging acceptability and information dissemination. The issue of trusted sources is reinforced by a recent evaluation study of 26 free-download Chinese infant and young child feeding apps developed by commercial entities [33].

As text messaging has the minimal requirement of a mobile phone, it can be readily expanded to many health care services and regions of limited resources [34]. SMS text messaging has shown its value in filling gaps in delivering health care services in settings where personal contact is limited. However, as an innovative way of care delivery, SMS text messaging intervention should not simply replace other forms of interventions, especially those with direct face-to-face contact, such as antenatal clinics and consultation with maternal and child health professionals.

The results also revealed several areas for improvement and lessons learned. It appears that the study did not always meet some mothers’ expectations, such as the need to respond immediately to their text inquiries. Clear communication upfront with the mothers is a way to manage such expectations. Although the SMS text messages were tailored to different stages of the child’s development, it was difficult to meet all specific needs of all users. The requirement for more personalized support is also advocated in the contemporary mHealth literature [35]. This might be achieved through the design of message texts based on participant’s detailed individual data, as has been done in a healthy lifestyle promotion project [36]. However, this undoubtedly will need more time and human resources. The time commitment required from the research team has also been highlighted by others [37]. The quality of telecommunication service might affect the SMS text message delivery, which, in turn, could affect the effectiveness of the intervention. Therefore, monitoring the delivery of the intervention should be considered.

For future research of using mHealth to improve infant and young child feeding, implementation research is needed to explore the facilitators and barriers before upscaling the intervention. As smartphone users have increased rapidly in recent years, there are opportunities to develop an evidence-based infant feeding app. The cost-effectiveness analysis of SMS text messaging interventions, or mHealth interventions in general, which is understudied, should be enhanced [34,38]. Finally, mHealth interventions should be based on health promotion theory to improve the likelihood of being effective [39,40].

Leveraging mobile phone technology promises to overcome barriers to accessing health systems, service delivery, and improved health promotion. Systematically documenting the planning and development, implementation, and process evaluation of an intervention is useful for informing and improving the development of future mHealth interventions.

## Abbreviations

BF: breastfeeding
CHC: community health center
EBF: exclusive breastfeeding
mHealth: mobile health
SMS: short message service
WHO: World Health Organization
